# Accidental Jorge Lobo's disease in a worker dealing with *Lacazia loboi *infected mice: a case report

**DOI:** 10.1186/1752-1947-3-67

**Published:** 2009-02-16

**Authors:** Patrícia Sammarco Rosa, Cleverson Teixeira Soares, Andréa de Faria Fernandes Belone, Raquel Vilela, Somei Ura, Milton Cury Filho, Leonel Mendoza

**Affiliations:** 1 Instituto Lauro de Souza Lima, Bauru, SP, Brazil; 2 Biomedical Diagnostic Laboratory Program, Department of Microbiology and Molecular Genetics, Michigan State University, East Lansing, MI, USA

## Abstract

**Introduction:**

Jorge Lobo's disease (Lacaziosis) is a subcutaneous infection of humans living in the Amazon region of Latin America, and in dolphins inhabiting the east coastal areas of the United States. The disease mainly affects people from rural areas living or working in close contact with vegetation and aquatic environments. Most patients refer having developed lesions after accidental trauma with plant thorns or insect bites. Inter-human transmission has never been confirmed suggesting that *Lacazia loboi *is acquired from environmental propagules.

**Case presentation:**

We report the case of a 41-year-old woman from São Paulo, Brazil, a non-endemic area of Jorge Lobo's disease, with *L. loboi *skin infection most likely accidentally acquired while manipulating experimentally infected mice in the laboratory.

**Conclusion:**

Because many patients with Jorge Lobo's disease do not recall accidental skin trauma before their infections, the possibility of accidentally acquired Jorge Lobo's disease through unnoticed broken skin should be considered during the clinical investigation of nodular skin diseases in people who have contact with the fungus or who live in endemic areas. This is the second report of animal to human transmission of this disease.

## Introduction

Jorge Lobo's disease is a chronic subcutaneous mycosis restricted to the geographic area of the Amazon (Brazil, Ecuador, Venezuela, Guyana, Suriname, Bolivia, Peru and Colombia) and other Latin American countries where isolated cases have also been reported [1-3]. The geographical distribution of Jorge Lobo's disease expanded after reports of the occurrence of the disease in dolphins [4]. Due to the fact that this anomalous pathogen resists culture, the reservoir of *Lacazia loboi *in nature is largely unknown. However, it is believed that *L. loboi *might be present in the humid areas of the Amazon basin [1,2,5].

The disease mainly affects male patients from rural areas living or working in close contact with vegetation and aquatic environments [1,5,6]. Most patients report having developed lesions after accidental trauma with plant thorns or insect bites, yet others do not recall trauma before the disease. The transmission between humans, especially domiciliary dissemination, has never been confirmed suggesting that *L. loboi *is mostly acquired from environmental propagules [1]. This hypothesis is strongly supported by the unusual disappearance of the disease when an entire Brazilian Caibi Indian tribe, usually affected by *L. loboi*, was relocated to a non-endemic area of the disease [1,6]. However, human to human, animal to animal and animal to human transmission cannot be ruled out since accidental and experimental Jorge Lobo's disease has been well documented [7-10].

## Case presentation

In March 2007, an otherwise healthy 41-year-old Caucasian female veterinarian from Instituto Lauro de Souza Lima, Bauru, São Paulo, Brazil complained of a slowly growing subcutaneous nodule on the inner side of her left hand middle finger. The patient did not recall any previous trauma in that particular anatomical area. The nodule had appeared 10 months earlier as a small hard cutaneous swelling on the proximal articular side of the middle phalanx, resembling a synovial cyst. The nodular skin lesion was very small and painless, therefore the patient did not seek immediate medical attention. In the following months after she had first noted the tissue swelling, the nodule increased in size and interfered with flexion of the affected finger. It was difficult to determine whether the nodule was attached to the skin or to the subcutaneous tissue at palpation. Clinical examination of the subcutaneous nodular lesion (~2.0 × 1.5 cm in diameter) by a surgeon led to diagnosis of a giant cell tumor of the flexor tendon, and surgical excision was advised. Physical examination revealed that the patient was in good general health and had no other similar skin lesions. Surgery was performed 10 months after the initial onset.

**Figure 1:**
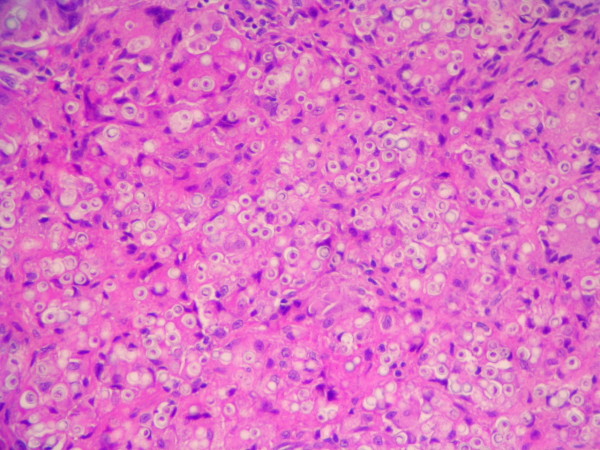
**Hematoxylin-eosin stained section of the biopsied tissue**. Numerous *Lacazia loboi *yeast-like cells are observed inside a granulomatous infiltrate (200×).

**Figure 2:**
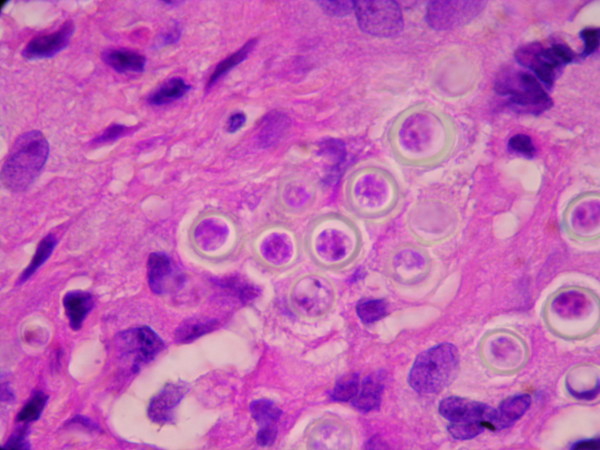
**The insert in the lower section is an enlargement showing *L. loboi *yeast-like cells in chains (1000×)**. Note the staining of the cytoplasmic content, an indication of viable cells.

**Figure 3:**
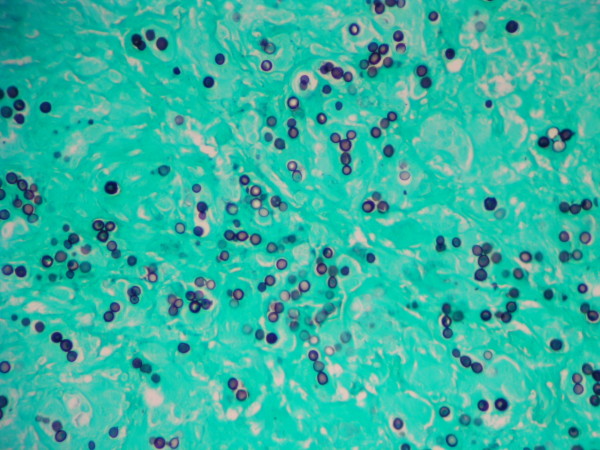
**Methenamine silver stained section of the same biopsied tissue as in Figure 1 showing the typical phenotypic features of *L. loboi *(200×)**.

**Figure 4:**
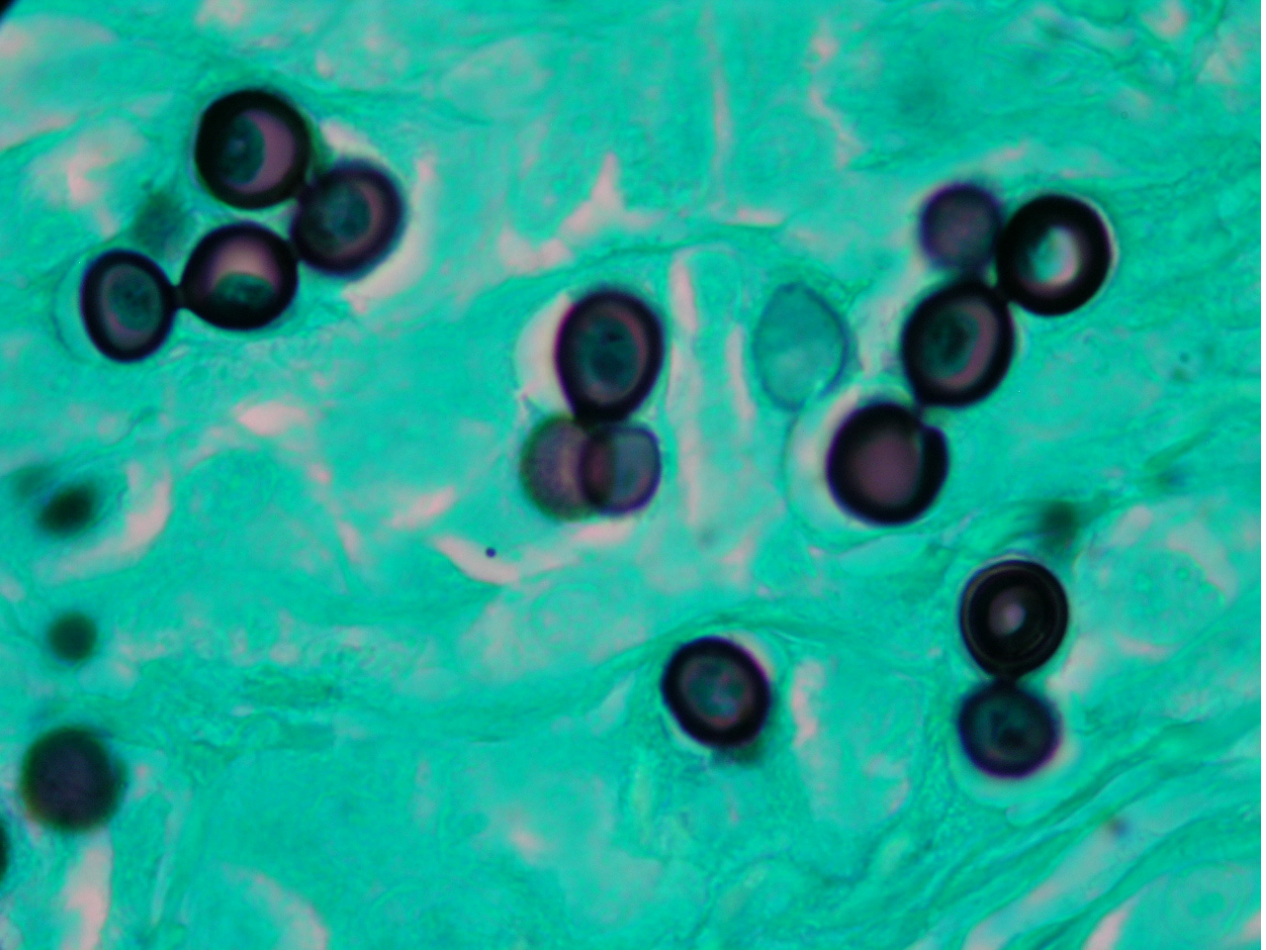
**The insert in the lower section is an enlargement depicting yeast-like cells connected by slender tubes (1000×)**.

The granulomatous 2 cm × 0.5 cm × 0.5 cm excised mass was attached to the dermis, nerves and tendons of the affected finger. It consisted of a firm yellowish tumoral-like mass resembling a lipoma, with a smooth bright surface. Because of the initial diagnosis of a benign tumor, microbiological testing (including culture) was not requested. Histopathological examination of hematoxylin-eosin stained sections showed a granulomatous infiltrate constituted by histiocytes and giant cells filled with numerous thick walled yeast-like cells, either singly or in chains, characteristic of *L. loboi *(Figures 1 and 2). The majority of the fungal elements in the infected tissues showed clear cytoplasmic content, a morphological characteristic of viable *L. loboi *yeast-like cells. Methenamine silver staining showed spherical to oval yeast-like cells mostly uniform in size and arranged singly or in small chains of cells linked by small tube-like structures (Figures 3 and 4). In respect to treatment, it has been observed that after use of clofazimine and dapsone, which have antimicrobial as well as anti-inflammatory activity, in concomitant leprosy and Jorge Lobo's disease patients, Jorge Lobo's lesions became atrophic. Itraconazole was chosen as the antifungal drug because of its low toxicity, high affinity to skin and good results when used with clofazimine in a Jorge Lobo's disease case. To prevent recurrence of the lesion in the present patient, drug therapy with clofazimine (50 mg/day), dapsone (100 mg/day) and itraconazole (200 mg/day) was initiated immediately after surgical intervention and continued for 1 year.

## Discussion

Our patient had lived for the past 10 years in the city of Bauru, São Paulo State, Brazil, a non-endemic geographical area for Jorge Lobo's disease. However, she had worked extensively with the fungus *L. loboi *in experimentally infected mice and had visited an endemic area for Jorge Lobo's disease. Her main laboratory activities included processing of human skin biopsies and mice foot pads infected with *L. loboi*. She also purified *L. loboi *fungal cells for mice inoculation, antigen preparation and *L. loboi *DNA extraction. Moreover, since *L. loboi *cannot be cultured, she had worked with purified live fungal yeast cells of *L. loboi *for maintenance of these strains in laboratory mice (she usually processed samples containing 5.1 × 10^6 ^*L. loboi *yeast-like cells). For the past 3 years, she had made several 1-week field trips to the State of Acre, Brazil. During these trips, she collected several skin biopsies from patients with the disease, performed viability tests and collected environmental samples from the tropical rainforest in the Antimary Reservation Area, where many Jorge Lobo's disease patients reside.

The human Jorge Lobo's disease cases reported in the literature refer to long-term incubation and slow growth of lesions in cases acquired from endemic areas [1,5]. However, the incubation intervals of humans residing outside the endemic areas of Jorge Lobo's disease varied. For instance, a French aquarium caretaker developed the disease 3 months after handling a *L. loboi *infected dolphin [10]. In contrast, in a man who apparently acquired the infection after traveling to Venezuela, the lesion appeared two and a half years after his trip to the endemic area [11]. A Canadian woman developed Jorge Lobo's disease 1 year after she had been to Guyana and Venezuela [12]. A bizarre case of experimental human Jorge Lobo's disease in a laboratory assistant inoculated with the yeast-like cells collected from a Venezuelan man with Jorge Lobo's disease was reported by Borelli [8]. The lesion slowly increased in size, and after 4 years, had attained 33 mm in diameter. In addition, experimental inoculation of BALB/c mice with *L. loboi *cells obtained from patients with the disease showed macroscopic lesions in 7 to 8 months [9]. Interestingly, it has been noted that lesions developed faster within 4 months after inoculation, on continuous passages from mice to mice, indicating a better adapted *L. loboi *to experimental mice infection [7].

In this case report, the patient had had contact with the fungus for about 10 years and her finger lesion increased in size relatively rapidly in an 8-month period since May 2006, when she first noted a small skin lesion. If the patient had acquired the infection from environmental propagules or by yeast-like cells from infected humans, most likely the fungus would slowly reproduce and the lesion would appear not in a few months, but years after the traumatic implantation, as is usually the case in patients with Jorge Lobo's disease [1,5,6]. Moreover, our patient developed a single lesion on her left hand middle finger extensively used to manipulate biopsied tissues and to inoculated mice with live yeast-like cells. This observation and the rapid progress of her finger lesion might suggest that she probably came into contact with *L. loboi *while manipulating samples from Jorge Lobo's disease patients or during experimental inoculation of mice, and less likely from natural environmental propagules of *L. loboi*.

This accidentally acquired case of Jorge Lobo's disease in a woman working with live *L. loboi *yeast-like cells raises several questions regarding the epidemiology and virulence of *L. loboi*. This fungus does not grow *in vitro *and it has never been identified in environmental samples. It is therefore believed to be a restricted human and dolphin pathogen, and transmission between susceptible hosts seems to be its survival strategy [6]. However, several lines of evidence suggest that *L. loboi *is acquired either through contact with propagules present in contaminated ecological niches closely related to rivers and damp wooded areas [1,2,5,6,11,12], or through contact with propagules from hosts infected with Jorge Lobo's disease (humans, dolphins and experimentally infected mice) [7,9,10]. The classical examples of naturally acquired Jorge Lobo's disease are the cases of the disease reported during trips to endemic countries [11,12] and the relocation of a Brazilian Indian tribe, where Jorge Lobo's disease cases were known, to a non-endemic area [1,6]. Alternatively, Jorge Lobo's disease could be directly acquired between hosts with the disease such as dolphins to humans [10] and by the many reports of experimental inoculation with live yeast-like cells of *L. loboi *in humans [8] and mice [7,9].

A classical myth about *L. loboi *is that this anomalous fungal pathogen has low virulence and is limited to the cool areas of the subcutaneous tissues [2,5]. However, the present report and other similar cases of naturally and experimentally acquired Jorge Lobo's disease [8-12] suggest that *L. loboi *has a well developed degree of virulence and can cause disease in apparently healthy as well as in immunocompromised hosts [3,8,10-13]. In the present report, the infected patient did not recall a major trauma at the site of infection. This implies that *L. loboi *could eventually reach the subcutaneous tissues through imperceptible abrasions on the upper layers of the skin. Since this pathogen is a slow growing fungus in its parasitic stage, *L. loboi *should possess a yet to be described adhesive mechanism to maintain close attachment to the injured skin. The activation of such a mechanism should be of particular importance in anatomical areas such as the hands, constantly washed with detergents and other chemicals.

Since *L. loboi *has been phylogenetically linked to other dimorphic fungal pathogens in the family Ajellomycetaceae [14,15], it could well be a dimorphic fungus with a mycelial form in nature and high tropism for soil of damp environmental areas. Thus, it could be acquired through skin trauma either by propagules accessible in nature, perhaps similar to that present in the mycelial form of *Paracoccidioides brasiliensis*, or by contact with the *L. loboi *yeast-like cells present in the host's infected tissues. Based on the epidemiology and location of the lesion in our patient, we also believe that *L. loboi *possess a sophisticated mechanism to remain attached to the injured skin, and therefore this adhesive substance might be an important virulence factor.

All in all, we believe that *L. loboi *has evolved and developed unique virulence factors allowing the pathogen to remain in the infected tissues for long periods of time (≤ 50 years), and thus becoming the perfect pathogen. This is in direct contrast to other members of the Ajellomycetaceae. The ability of *L. loboi *to remain in the infected tissues for years without killing the host might have had a significant role in shaping its genome during its evolutionary path to a more restricted mammalian pathogen.

## Conclusion

This is a report of animal to human transmission of Jorge Lobo's disease. Because most patients with Jorge Lobo's disease do not recall accidental skin trauma during daily activities before their infections, the possibility of having accidentally acquired Jorge Lobo's disease through unnoticed broken skin on people residing and/or working in endemic areas, health care personnel dealing with Jorge Lobo's disease proven cases in humans or dolphins, or researchers working with purified yeast-like cells of *L. loboi*, should be carefully considered during clinical investigation of nodular skin disease.

## Consent

Written informed consent was obtained from the patient for publication of this case report and any accompanying images. A copy of the written consent is available for review by the Editor-in-Chief of this journal.

## Competing interests

The authors declare that they have no competing interests.

## Authors' contributions

PSR and LM contributed to the study concept and drafting of the manuscript. MCF and SU were responsible for patient management. CTS was responsible for the histopathological diagnosis. CTS, AFFB and RV undertook the medical literature search and critical review of the manuscript. All authors read and approved the final manuscript.
